# The paradoxes of the Late Hesperian Mars ocean

**DOI:** 10.1038/s41598-019-42030-2

**Published:** 2019-04-05

**Authors:** M. Turbet, F. Forget

**Affiliations:** Laboratoire de Météorologie Dynamique, IPSL, Sorbonne Universités, UPMC Univ Paris 06, CNRS, 4 place Jussieu, 75005 Paris, France

## Abstract

The long-standing debate on the existence of ancient oceans on Mars has been recently revived by evidence for tsunami resurfacing events that date from the Late Hesperian geological era. It has been argued that these tsunami events originated from the impact of large meteorites on a deglaciated or nearly deglaciated ocean present in the northern hemisphere of Mars. Here we show that the presence of such a late ocean faces a paradox. If cold, the ocean should have been entirely frozen shortly after its formation, thus preventing the formation of tsunami events. If warm, the ice-free ocean should have produced fluvial erosion of Hesperian Mars terrains much more extensively than previously reported. To solve this apparent paradox, we suggest a list of possible tests and scenarios that could help to reconcile constraints from climate models with tsunami hypothesis. These scenarios could be tested in future dedicated studies.

## Introduction

The existence of liquid water oceans on ancient Mars has long been a topic of debate^1–6^ and has strong implications for the search for life in the solar system. Specifically, the lack of wave-cut paleoshore-line features and the presence of lobate margins seem to be inconsistent with the presence of a Late Hesperian ocean (see^7^ and references therein). A review of the historical Late Hesperian ocean controversy, as well as alternative scenarios to explain geologic observations are proposed in^6^. Recently, two studies^7,8^ independently identified the presence of highland-facing lobate debris deposits in Arabia Terra, along the dichotomy boundary, interpreted as tsunami deposits. The overlap of several distinct lobate deposits as well as their wide range of elevation suggest the possibility of multiple (at least two) tsunami events^7,8^. Both studies^7,8^ reported that these tsunami events were likely caused by the collision of large meteorites (3–6 km in diameter) on an ice-free or sea-ice covered ocean located in the northern lowlands of Mars.

Therefore, Mars could have hosted a large body of liquid water hundreds of millions of years later than the formation of the valley networks. Below we discuss the implications of the presence of a deglaciated (or nearly deglaciated) ocean on both the atmosphere and the geology of Late Hesperian Mars.

## The paradoxes of a cold ocean

Sustaining a liquid-water ocean, even ice-covered, would require a very strong greenhouse effect involving a mixture of greenhouse gases. 3-D climate modeling of early Mars under an atmosphere composition of only CO_2_ and H_2_O, performed with a water cycle that includes water vapor and clouds, is unable to maintain significant amount of liquid water anywhere on the red planet, even when maximizing the greenhouse effect of H_2_O and CO_2_ ice clouds^9,10^. This major result holds independently of the specific CO_2_ atmospheric content, water content and obliquity. More specifically, an initially warm northern ocean possibly fed by outflow channel formation events should freeze extremely rapidly under a Late Hesperian Mars CO_2_ atmosphere^11,12^. For instance, a 303 K, 200m deep ocean (under a 0.2 bar CO_2_ atmosphere, at 45° obliquity) would become entirely ice-covered after ∼1 martian year, and frozen solid after ∼4 × 10^3^ martian years^12^. More generally, a northern plains ocean would completely freeze within 10^4^ years, whatever the obliquity, surface pressure of CO_2_, and whatever the initial size and temperature of the ocean assumed^12^. The freezing process is particularly efficient at low obliquity (because of the low insolation at the North Pole) and low CO_2_ atmospheric pressure (because of the small greenhouse effect of CO_2_). At high obliquity and high CO_2_ surface pressures, the poles are warmer, but still too cold to sustain a liquid water ocean (even with ice cover)^12^.

**Figure 1:**
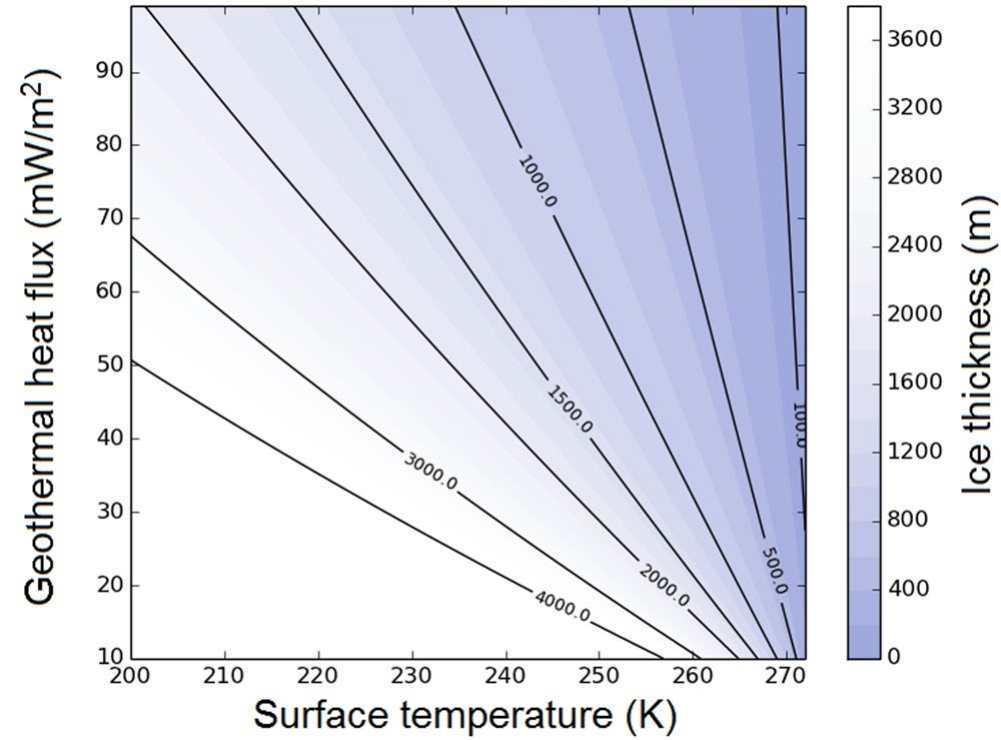
Presents the minimum ice thickness of the ocean calculated as a function of the temperature at the top of the sea ice cover and of the geothermal heat flux.

To avoid the need of additional hypothetical greenhouse gases (see below), it is tempting to speculate that the ocean could have remained liquid below a protective ice layer, thanks to a strong geothermal heat flux. We calculated the minimum ice thickness (see Methods) required to sustain subsurface liquid water, as shown in Fig. 1. Even for a geothermal heat flux of 80 mW m^−2^ (which is an upper limit on the geothermal heat flux expected during the Late Hesperian era, and for volcanically active regions^13^), we found that the ice thickness grows very rapidly as the surface temperature drops below freezing. The range of conditions that would have allowed a long-term ice-covered ocean is very narrow: for instance, to limit the growth of the ice thickness to 100 m or less, the annual mean surface temperature must range between 270 and 273 K. As a comparison, the minimum ice thickness is ∼1 km for an annual mean surface temperature of 240 K (see Fig. 1) which corresponds to the maximum annual mean temperature predicted in the northern lowlands by 3-D numerical climate models^10,12^. The presence of salts would have depressed the ocean’s water triple point. However, even for a drop of 21 K (the lowest freezing point obtainable for a NaCl brine is 252 K^14^), we calculated that the minimum ice thickness is ∼0.5 km for an annual mean surface temperature of 240 K.

Even with a frozen surface, a Northern ocean on Mars would have been unstable on geological timescales. If one assumes that a water ocean is formed during a short period of time (e.g. by catastrophic outflows) and then left on its own, calculations show that it would have eventually disappeared after ∼10^5^ martian years because the water would have been progressively transported toward the elevated regions of Mars through sublimation and subsequent adiabatic cooling and condensation^10,12^. Only a thick lag deposit of silicate material^15,16^ formed on a permanently frozen surface could have prevented the water from getting sublimated and migrating away to the elevated terrains of Mars. However, even then, it seems almost unavoidable that the ocean would have frozen down to its bottom, as hypothesized in the scenarios where the Vastitas Borealis Formation (VBF) is thought to be a remnant of the sublimation residue of a Late Hesperian ocean^4,11,16^.

## The paradoxes of a warm ocean

Alternatively, we could imagine that for some time the Late Hesperian Martian climate was sufficiently warmed by additional strong greenhouse gases, and thus keeping the ocean at least partly liquid. For instance, reducing gases (e.g. CH_4_ and H_2_) offer a way to warm the surface of ancient Mars above the melting point of water^17–21^. This effect results in part from the strong collision-induced absorptions of CO_2_-CH_4_, CO_2_-H_2_ and H_2_-H_2_ pairs^17,18,22^.

However, the persistence of a deglaciated ocean during the Late Hesperian on Mars would raise several issues. New 3-D Global Climate simulations (see Methods) confirm that a deglaciated northern ocean could be permanently sustained with the assumption of enough greenhouse gases (CO_2_ and H_2_ here). However, ocean waters would evaporate rapidly and subsequently migrate toward the elevated Martian terrains through the mechanism of adiabatic cooling mentioned above for the case of a frozen ocean. This process is rapid because evaporation and sublimation rates increase exponentially with temperature. For instance, a 200 m deep deglaciated northern ocean would completely evaporate within ∼10^3^ martian years, whatever the obliquity, surface pressure of CO_2_ and of additional reducing gases, and whatever the initial temperature (>273 K) of the ocean assumed.

**Figure 2:**
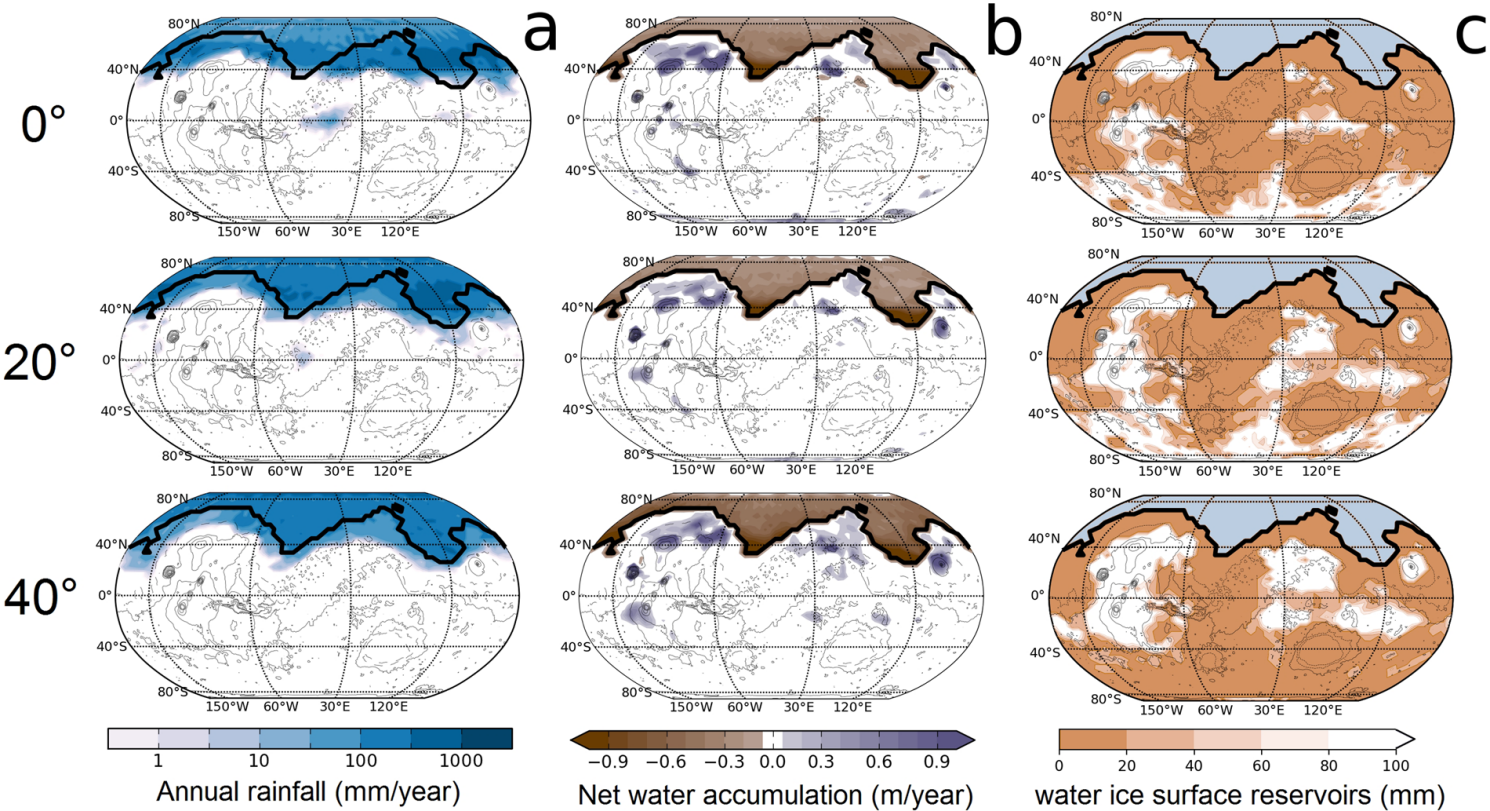
Presents the annual cumulated rainfall, the annual net surface accumulation of water, and the position of permanent ice reservoirs for various 3-D Global Climate simulations.

In the simulations, a part of the atmospheric water returns to the surface as rain, near the ocean shoreline (see Fig. 2a). Such precipitation would produce extensive fluvial erosion, in particular in the regions where evidence for tsunami events have been reported^7,8^, and as long as the deglaciated northern ocean remains. The remainder is sequestered as ice on the elevated terrains (see Fig. 2c). In any case, for the northern ocean to survive, an intense hydrological cycle had to occur in order to replenish the water that was transported to the elevated terrains. Although previous regional maps seem to indicate an absence of such a strong hydrological cycle in the mid and late Hesperian geological record (see^23^ and references therein), this prediction could be tested in more details through further high-resolution geological investigations, in particular along the proposed paleo-ocean shoreline.

To solve this paradox, one hypothesis could be for the ocean to be replenished by groundwater. In this scenario, water that condensed on the elevated Martian volcanic regions would have formed thick glaciers that would undergo melting at their base, possibly introducing the meltwater into subsurface aquifers^24^. These subsurface liquid water reservoirs could then have provided the water that carved the outflow channels, thus replenishing the northern ocean. Such an hypothesis would be consistent with our 3-D Global Climate simulations (see Fig. 2b) in which water tends to condense preferentially close to the regions that sourced the outflow channels. However it is difficult to reconcile this hypothesis with the estimated lifetime of the ocean. It has been reported that at least two large tsunami events were produced by bolide impacts, resulting in craters 30–50 km in diameter^7,8^. Based on the crater frequency rates of Rodriguez^7^, the rate of Late Hesperian marine impacts producing craters ∼30 km in diameter is one every 2.7 million years. Unless the tsunamis were the result of very unlikely occurrences, the ancient ocean would have to survive for a period of at least a few million years to produce the reported two consecutive tsunami events^7,8^. This is also supported by the detection of glacier valleys cross cuting first, older tsunami deposits and having floors partly covered by younger, second tsunami deposits, indicating that the time gap between the two tsunami events was geologically significant^7^. We estimate from our 3-D Global Climate simulations (see Fig. 2b) that the net evaporation rate of the ocean is at least 0.6 m per martian year, and that at least 10^4^ km^3^ of water would be required for replenishment per martian year in order for the ocean to remain stable. Thus, as much as 1.5 × 10^10^ km^3^ (e.g. ∼100 km GEL) of water would need to have flown through the outflow channels for a deglaciated northern ocean to survive for 2.7 million years. This amount is several orders of magnitude larger than previous estimates of the total amount of water required to erode all the Martian outflow channels^25^.

This paradox could be partly overcome if the Late Hesperian ocean was extremely briny^7,11,26^. First, salts would have depressed the ocean’s water triple point by tens of Kelvins, relaxing the constraint on the hypothetical greenhouse gases (assumed here to be CH_4_ and H_2_) concentration needed for the ocean to remain deglaciated. Next, reducing the ocean surface temperature would reduce the evaporation rate of the ocean, thus relaxing the constraint on the rate of replenishment needed for the ocean to survive millions of years. For a drop of 21 K in ocean surface temperature, the ocean evaporation rate^12^ could drop by a factor of ${e}^{-\frac{{L}_{{\rm{evap}}}{{\rm{M}}}_{{{\rm{H}}}_{2}{\rm{O}}}}{{\rm{R}}}(\frac{1}{\mathrm{252\ }K}-\frac{1}{\mathrm{273\ }K})}$ ∼ 5, with *L*_evap_ the latent heat of evaporation, ${{\rm{M}}}_{{{\rm{H}}}_{2}{\rm{O}}}$ the molar mass of water and R the ideal gas constant. Ultimately, precipitation (here snowfall) near the ocean shorelines would be too cold to melt, thus relaxing the constraint on the fluvial erosion. However, for this scenario to work, the salinity of the ocean would have to be extremely high, and as much as ∼2 × 10^6^ km^3^ of salts, i.e. ∼10 m GEL (Global Equivalent Layer) would have to be accounted for, whatever the nature of brine considered^14^. Although salts such as perchlorates have been detected *in situ* by the Phoenix^27^ and Viking landers^28^ at the ∼0.1% level, whether this is sufficient or not to account for the presence of ∼10 m GEL of salts is left for future investigations.

## Alternative solutions

Several hypothetical scenarios could potentially reconcile the tsunami hypothesis^7,8^ with the geological records and our understanding of the Martian climate.

In one class of scenarios, the ocean existed but it was fully (or almost fully) glaciated, and potentially protected by a lag deposit. Tsunami events could have been produced in response to consecutive meteoritic impacts, for instance resulting from a collision with the different pieces of a broken body like the Shoemaker-Levy 9 comet which hitted Jupiter in July 1994^29^. The first impact would (at least partially, and as a result of either (i) direct melting or (ii) impact-induced global climate change) deglaciate the ocean, and the following impacts could then produce the tsunami.

Tsunami events could also be produced in response to impact-triggered pressure waves propagating in a thin, deep subsurface ocean located below a km-thick cover of ice^30^. The same impact could also expel liquid water from the deep ocean, that would then form a huge flow on top of the ice cover. In principle, this scenario is compatible with the minimum ice cover thickness of ∼1 km (calculated above for the maximum annual mean temperature predicted in the northern lowlands by 3-D Global Climate models^10,12^) and the maximum depth of the hypothetical ocean^8^ estimated at ∼1.4 km.

In a second class of scenarios, there is no perennial ocean. Instead, the tsunami events could have been produced by the catastrophic outflow channel formation events that occurred in the same region and at the same epoch. The sudden release of extremely large amounts of water could produce large waves across the northern lowlands terrains, and potentially resurfacing them. A similar scenario was previously invoked to explain the formation of sedimentary deposits on the slopes of the northern lowlands^31^. For very large discharge rates (∼10^9^ m^3^ s^−1^), the mean flow velocity and depth^12^ can reach 10 m s^−1^ and 50 m, respectively, for a flow width of ∼2000 km, typical of the Northern lowlands characteristic horizontal extension. These numbers are of the same order of magnitude than those calculated for impact-generated tsunami events^7,8^. Although most recorded tsunami deposits do not face the circum-Chryse outflow channels^7^, these extreme water flows could have been guided in various directions by remnant ice deposits, originating either (i) from the freezing of water from previous outflows, or (ii) from atmospheric precipitation^12^.

The hypothetical alternative solutions mentioned here should be explored in greater detail with appropriate numerical models in the future.

## Methods

### Ice thickness calculation

The ice thickness can be estimated using the assumption that the transport of energy inside the water ice layer is controlled by conduction. The thermal conduction heat flux *F* can be written as follows:

1 $$F=\frac{A}{{h}_{{\rm{ice}}}}\,\mathrm{ln}(\frac{{T}_{{\rm{bottom}}}}{{T}_{{\rm{surf}}}})$$

with *λ*_ice_(*T*) = $\frac{A}{T}$ the thermal conductivity of ice (with *A* = 651 W m^−1^)^32^, *h*_ice_ the thickness of the water ice layer, *T*_surf_ the temperature at the top of the water ice layer and *T*_bottom_ the temperature at the bottom. At the interface between ice and liquid water, *T*_bottom_ is equal to 273,15 K. At equilibrium, the annual mean thermal conduction heat flux *F* is dominated by the geothermal heat flux *F*_geo_. This results in the expression:

2 $${h}_{{\rm{ice}}}=\frac{A}{{F}_{{\rm{geo}}}}\,\mathrm{ln}(\frac{273.15}{{T}_{{\rm{surf}}}})$$

### Global Climate Model simulations

We use the LMD Early Mars 3-D Global Climate Model^9,10,12,33^. The model includes both the water and CO_2_ cycle (condensation and sublimation on the surface and in the atmosphere; formation and transport of clouds; precipitation and evaporation). It also includes a detailed radiative transfer module adapted to a thick CO_2_-dominated atmosphere complemented with H_2_O and H_2_. We performed three numerical climate simulations at the obliquities 0°, 20° and 40°. Simulations were performed with a spatial resolution of 64 × 48 × 26 (in longitude × latitude × altitude), using the present-day Mars MOLA topography. An ocean was placed in the northern lowlands of Mars, at elevations lower than −3.9 km. This value was chosen to match the best case of the tsunami propagation scenarios of Costard^8^. We use a two layers slab-ocean model to treat the oceanic region^34^. The transport of heat by the ocean circulation is not taken into account here. We fixed the total atmospheric pressure to 1 bar (see^35^ and references therein), and varied the concentration of H_2_ (from 5 to 20%) in order to sustain a deglaciated ocean (annual mean temperature is around 276 K). The concentration of H_2_ has been chosen to ensure that the deglaciated ocean has the lowest possible temperature. This was achieved with concentrations of H_2_ equal to 7, 6 and 5 % for the simulations at 0, 20 and 40° obliquities, respectively.
